# A distributed geospatial approach to describe community characteristics for multisite studies

**DOI:** 10.1017/cts.2021.7

**Published:** 2021-02-05

**Authors:** Patrick H. Ryan, Cole Brokamp, Jeff Blossom, Nathan Lothrop, Rachel L. Miller, Paloma I. Beamer, Cynthia M. Visness, Antonella Zanobetti, Howard Andrews, Leonard B. Bacharier, Tina Hartert, Christine C. Johnson, Dennis Ownby, Robert F. Lemanske, Heike Gibson, Weeberb Requia, Brent Coull, Edward M. Zoratti, Anne L. Wright, Fernando D. Martinez, Christine M. Seroogy, James E. Gern, Diane R. Gold

**Affiliations:** 1 Department of Pediatrics, University of Cincinnati, College of Medicine, Cincinnati, OH, USA; 2 Division of Biostatistics and Epidemiology, Cincinnati Children’s Hospital Medical Center, Cincinnati, OH, USA; 3 Center for Geographic Analysis, Harvard University, Cambridge, MA, USA; 4 Asthma and Airways Disease Research Center, University of Arizona, Tucson, AZ, USA; 5 Department of Community, Environment, and Policy, Mel and Enic Zuckerman College of Public Health, University of Arizona, Tucson, AZ, USA; 6 Division of Clinical Immunology, Icahn School of Medicine at Mount Sinai, New York, NY, USA; 7 Rho, Inc., Chapel Hill, NC, USA; 8 Department of Environmental Health, Harvard T. H. Chan School of Public Health, Boston, MA, USA; 9 Department of Biostatistics, Mailman School of Public Health, Columbia University, New York, NY, USA; 10 Washington University School of Medicine, Division of Pediatric Allergy, Immunology, and Pulmonary Medicine, St. Louis, MO, USA; 11 Vanderbilt University School of Medicine, Division of Allergy, Pulmonary, and Critical Care Medicine, Nashville, TN, USA; 12 Henry Ford Health System, Detroit, MI, USA; 13 Division of Allergy and Immunology, Augusta University, Augusta, GA, USA; 14 Department of Pediatrics, University of Wisconsin School of Medicine and Public Health, Madison, WI, USA; 15 Division of Pulmonary and Sleep Medicine, Department of Pediatrics, College of Medicine, University of Arizona, Tucson, AZ, USA; 16 Channing Division of Network Medicine, Brigham and Women’s Hospital and Harvard Medical School, Boston, MA, USA

**Keywords:** Geospatial analysis, GIS, participant confidentiality, determinants of health, multisite studies

## Abstract

Understanding place-based contributors to health requires geographically and culturally diverse study populations, but sharing location data is a significant challenge to multisite studies. Here, we describe a standardized and reproducible method to perform geospatial analyses for multisite studies. Using census tract-level information, we created software for geocoding and geospatial data linkage that was distributed to a consortium of birth cohorts located throughout the USA. Individual sites performed geospatial linkages and returned tract-level information for 8810 children to a central site for analyses. Our generalizable approach demonstrates the feasibility of geospatial analyses across study sites to promote collaborative translational research.

## Introduction

Maintaining patient privacy is a common challenge faced by researchers seeking to understand the relationship of place and health [1–4]. This issue can be especially problematic in multisite studies due to study protocols and confidentiality concerns that limit the sharing of geographic data. Existing approaches to geospatial analyses in multisite studies include the use of a central site to conduct analyses or the application of spatial techniques (e.g. changing geographic coordinates to protect confidentiality, i.e. “geomasking”) to protect patient privacy [4]. However, the former method is limited by data use agreements (DUAs) and the latter may result in exposure misclassification when geomasked locations result in spatial misalignment.

Alternatively, study sites may perform geocoding and geospatial linkages independently before removing identifiable information for joint analyses. This decentralized approach, however, faces challenges in reproducibility and standardization due to geocoding methods, geographic information software (GIS), and expertise that varies across study sites. Here, we describe the application of a novel method to perform reproducible, standardized, and confidential geospatial analyses for multisite studies. Our approach extends a previously developed Decentralized Geomarker Assessment for Multi-site Studies (DeGAUSS) containerization platform to perform geocoding and extraction of polygon feature geospatial data over multiple time periods and large geographic areas [5]. As an example case, we use our approach to ascertaining US census tract-level information for participants enrolled in the Children’s Respiratory and Environmental Workgroup (CREW), a network of 12 birth cohorts each studying the development of allergy and asthma in childhood [6].

## Materials and Methods

### Study Population

Our approach was motivated by the CREW consortium, a network of birth cohorts recruited from 1980 to 2020. Information regarding participating cohorts, including eligibility criteria, study recruitment, and other methods, are published [6] and in Supplementary Table 1. Due to its large sample size and geographic distribution, CREW provides a unique platform to examine environmental and community factors that contribute to the disproportionately higher burden of asthma-related morbidity and mortality among disadvantaged communities [7–10]. A CREW data sharing protocol and DUA were approved by the local IRB for each cohort. However, the DUA allowed only limited datasets to be shared and prohibited the distribution of identifying information, including addresses or geocodes.

### Distributed Geospatial Analysis

We extended our DeGAUSS software to enable all CREW sites, including those with limited geospatial expertise, to derive spatio-temporal US census tract-level information for their participants at birth. A key advantage to DeGAUSS is the use of a software containerization platform to wrap necessary software, system dependencies, and geospatial data in a stand-alone package that will work the same regardless of its host environment [11]. Previously, we created DeGUASS and applied this tool in the Electronic Medical Records and Genomics (eMERGE) network in a proof-of-concept study [5]. Whereas our prior DeGAUSS software included only a geocoder and code to link geospatial coordinates to nearby roadways and one census tract variable, the CREW consortium required significantly expanded geographical and temporal data to be included for analyses. Therefore, a new custom DeGAUSS container containing decennial US Census data (described below), census tract polygon boundary files for the 1980, 1990, 2000, and 2010 census, and R code (R Foundation for Statistical Computing: Vienna, Austria; 2014) was created to merge census tract-level data to the geocoded locations of CREW birth addresses. Additional details regarding DeGAUSS and the CREW container are provided in the supplementary materials and online [12,13].

**Fig. 1.**
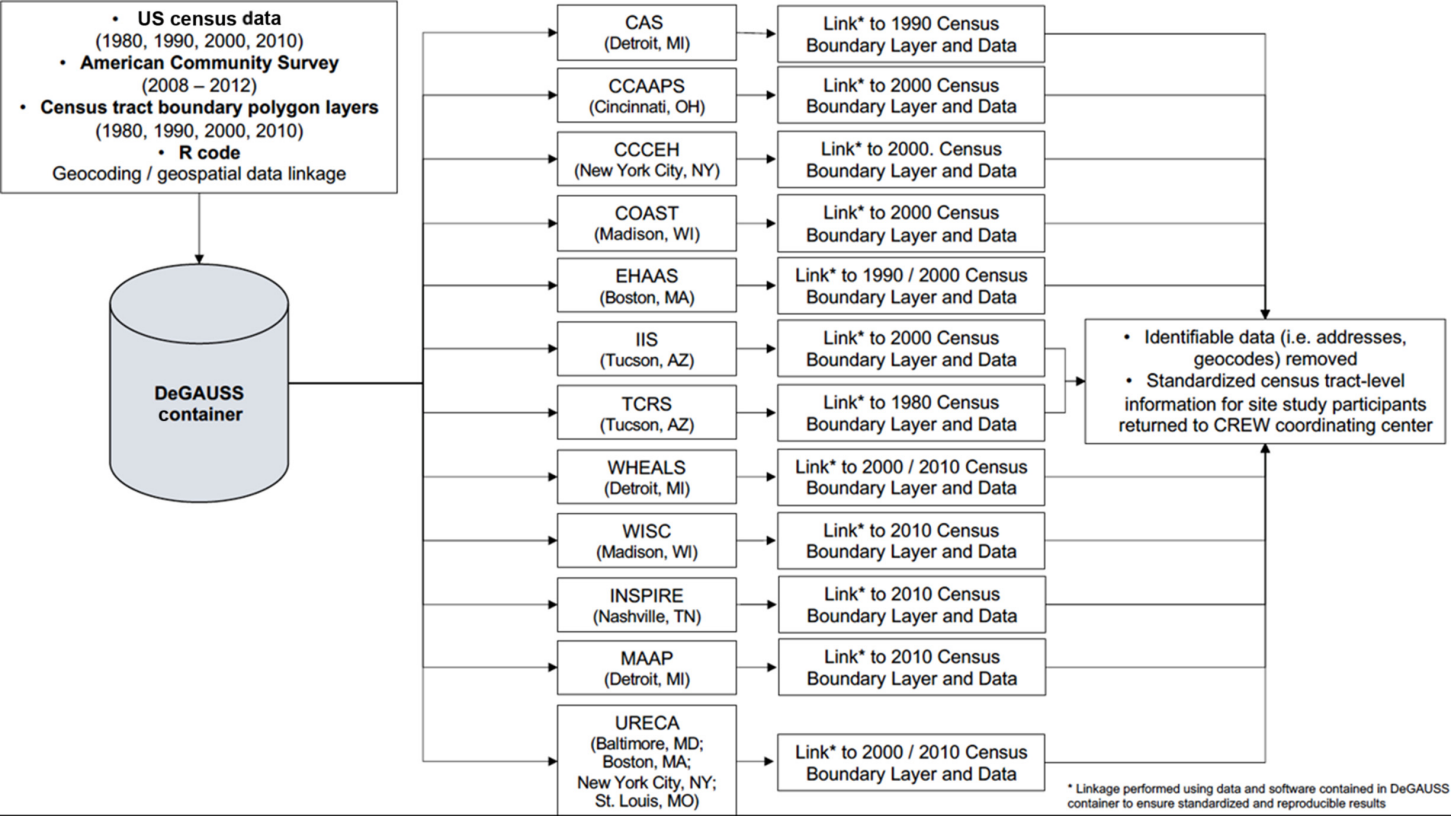
Flow diagram of distributed geospatial analyses for the Children’s Respiratory and Environmental Workgroup (CREW) consortium. CAS, Childhood Allergy and Asthma Study; COAST, Childhood Origins of Asthma Study; CCAAPS, Cincinnati Childhood Allergy and Air Pollution Study; CCCEH, Columbia Center for Children’s Environmental Health; EHAAS, Epidemiology of Home Allergens and Asthma Study; INSPIRE, Infant Susceptibility to Pulmonary Infections and Asthma Following RSV Exposure; IIS, Infant Immune Study; MAAP, Microbes, Allergy, Asthma, and Pets; TCRS, Tucson Children’s Respiratory Study; URECA, Urban Environment and Childhood Asthma; WHEALS, Wayne County Health, Environment, Allergy, and Asthma Longitudinal Study; WISC, Wisconsin Infant Study Cohort.

A flow diagram depicting the distributed approach to geospatial analyses for the CREW consortium is provided in Figure 1. The DeGAUSS container image was created by C.B. at a single location and distributed to each cohort. The DeGAUSS software required cohort users to provide an input.csv file containing the geocoded coordinates of their participants’ birth record address. Site end users also specified the census year to assign the appropriate tract boundary file and census data based on participants’ year of birth. The output data file from the DeGAUSS container contained the original input data, including geocoded locations, and appended census tract information including the census tract FIPS code in which the birth record address was located and census variables. Site end users manually removed identifying information, including the geographic coordinates and census tract FIPS code, prior to returning the de-identified dataset to a central coordinating center.

### US Census/American Community Survey Data

Longitudinal US Census data and boundary files for the years 1980, 1990, 2000, and 2010 were downloaded for the entire USA from Social Explorer (www.socialexplorer.com, New York City, NY: Social Explorer 2017; accessed 12/17–1/18) by year, requested variables, and tract level of geography. For 1980, certain census variables were only available from the National Historic GIS data service (Minneapolis, MN: NHGIS; accessed 11/17–1/18) and were updated to reflect variable calculation definitions used in later censuses. As the 2010 census did not include information regarding median household income, median gross rent, or median housing values, these data were downloaded from the 2008–2012 American Community Survey (ACS, https://www.census.gov/programs-surveys/acs/data.html; accessed 12/17–1/18). Additional information regarding the census and ACS variables downloaded and included in the DeGAUSS container for linkage to birth record addresses is available in the supplementary material.

### Statistical Analyses

After sharing de-identified data with a central site, descriptive statistics and box-and-whisker plots for all census tract variables were calculated for the combined CREW consortium and for each cohort individually. Comparison of census tract-level to self-reported race, ethnicity, and household income was conducted by plotting the distribution of each census tract-level variable according to self-reported variable. Self-reported household income was compared to census tract median household income using each cohorts’ income categories as collected by questionnaire. Additional details regarding self-reported race, ethnicity, and income information are available in the supplementary material.

## Results

All cohorts (*n* = 12) completed the distributed analysis and returned de-identified data to the coordinating center. Collectively, 8997 participants were enrolled in CREW cohorts, and 98% (*n* = 8810) of these had birth record addresses geocoded with sufficient precision for linkage to a census tract.

**Fig. 2.**
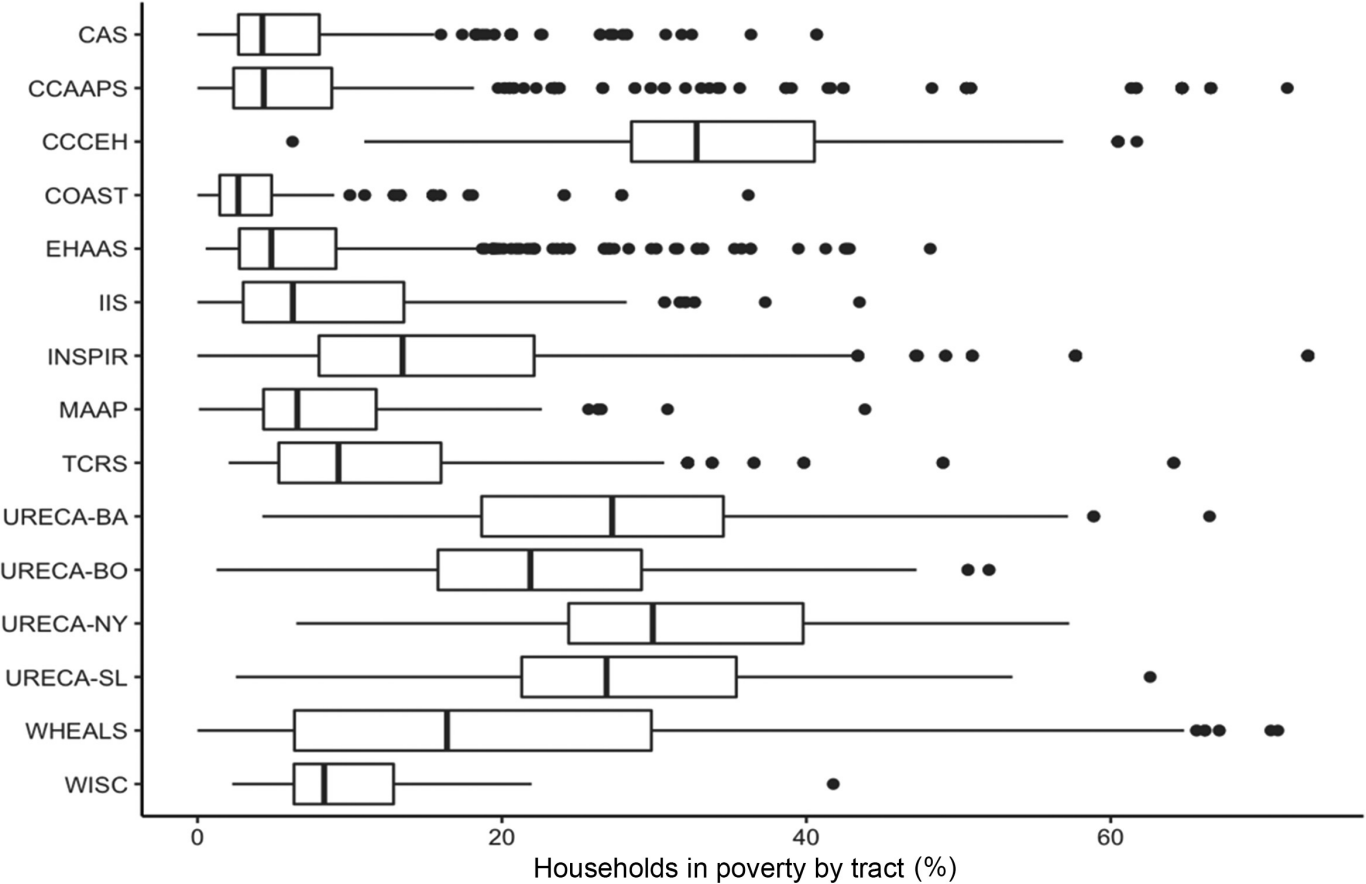
Percentage* of household poverty in census tacts where CREW participants resided at birth. CAS, Childhood Allergy and Asthma Study; COAST, Childhood Origins of Asthma Study; CCAAPS, Cincinnati Childhood Allergy and Air Pollution Study; CCCEH, Columbia Center for Children’s Environmental Health; EHAAS, Epidemiology of Home Allergens and Asthma Study; INSPIRE, Infant Susceptibility to Pulmonary Infections and Asthma Following RSV Exposure; IIS, Infant Immune Study; MAAP, Microbes, Allergy, Asthma, and Pets; TCRS, Tucson Children’s Respiratory Study; URECA, Urban Environment and Childhood Asthma (BA, Baltimore; BO, Boston; NY, New York; SL, St. Louis), WHEALS, Wayne County Health, Environment, Allergy, and Asthma Longitudinal Study; WISC, Wisconsin Infant Study Cohort.

A summary of population, race, ethnicity, and income data for the census tracts in which participants resided at birth is provided in Table 1. CREW participants resided in both low and high population density regions as reflected in the average tract population density (persons per km^2^) that ranged from 148 for Wisconsin Infant Study Cohort (WISC) participants in rural Wisconsin to 45,772 for CCCEH participants in New York City (Table 1). Overall, CREW participants lived in tracts that were 67% White, 23% Black, 2% Asian, and 9% Other race. There was, however, variability in tract racial distribution both within and across cohorts (Table 1); eight cohorts enrolled participants from tracts where more than 75% of the population was White, while participants enrolled in URECA, WHEALS, and CCCEH resided in census tracts with populations having a greater proportion of Black or Other race. Most cohorts enrolled participants from tracts having a Hispanic population less than 10%, though the mean Hispanic population in tracts of CCCEH, IIS, TCRS, and URECA (Boston, MA and New York, NY sites) participants ranged from 21 to 57%. The overall mean of households in poverty was 15% in census tracts where CREW participants resided at birth but as shown in Figure 2, there was significant variability within and across cohorts. Additional information on census tract-level median household income, percentage (%) of households in poverty, % occupied housing, and median housing value as indicators of neighborhood income and housing is provided in the supplementary materials.

**Table 1.** Summary of census tract-level population, race, ethnicity, and income for CREW participants at birth record address

			Census variable (mean, SD)							
			Population	Race				Ethnicity	Income	
Cohort	Participants with deocoded birth address	Census year	Population density (/km^2^)	% White	% Black	% Asian	% Other	% Hispanic	Median household income (2012 USD)	% Below poverty level
CREW	8810	1980/1990/2000/2010	5734 (13,897)	67 (34)	23 (32)	2 (3)	9 (14)	13 (21)	53,930 (26,368)	15 (14)
CAS	825	1990	1457 (970)	93 (13)	4 (13)	2 (2)	1 (1)	1 (1)	73,449 (26,708)	6 (5)
CCAAPS	762	2000	1346 (938)	79 (28)	17 (28)	1 (2)	2 (1)	1 (1)	64,895 (27,632)	10 (15)
CCCEH	724	2000	45,772 (1956)	16 (9)	41 (28)	2 (2)	42 (21)	57 (27)	31,171 (7308)	34 (9)
COAST	280	2000	877 (882)	89 (11)	4 (5)	3 (6)	4 (3)	4 (4)	71,090 (20,443)	5 (6)
EHAAS	499	1990/2000	3944 (3936)	83 (23)	9 (19)	5 (4)	3 (6)	5 (7)	86,218 (33,980)	8 (8)
IIS	481	2000	1380 (880)	79 (12)	4 (2)	3 (1)	15 (11)	23 (17)	57,774 (23,415)	9 (8)
INSPIRE	1921	2010	628 (692)	73 (25)	20 (23)	2 (2)	6 (5)	7 (7)	47,606 (20,237)	16 (12)
MAAP	141	2010	1165 (700)	82 (19)	11 (17)	4 (5)	3 (2)	4 (3)	69,042 (27,253)	9 (7)
TCRS	1142	1980	1172 (813)	85 (14)	3(4)	1 (1)	10 (12)	21 (21)	51,535 (17,849)	12 (8)
URECA - Baltimore	163	2000/2010	6480 (3468)	16 (22)	79 (26)	1 (2)	4 (5)	3 (7)	33,202 (12,999)	28 (13)
URECA – Boston	143	2000/2010	8244 (4544)	30 (26)	46 (29)	5 (6)	20 (11)	25 (17)	44,680 (20,190)	23 (11)
URECA - New York	118	2000/2010	33,137 (14,409)	26 (15)	41 (19)	3 (3)	30 (13)	51 (20)	30,967 (17,645)	31 (12)
URECA - St. Louis	178	2000/2010	2375 (1292)	21 (25)	76 (27)	1 (2)	3 (8)	2 (3)	31,467 (10,297)	28 (11)
WHEALS	1248	2000/2010	2376 (1198)	41 (38)	53 (41)	2 (4)	5 (6)	4 (10)	49,092 (24,995)	20 (15)
WISC	185	2010	148 (301)	96 (3)	1 (1)	1 (1)	3 (3)	3 (3)	50,871 (9775)	9 (5)

CREW, Children’s Respiratory and Environmental Workgroup; CAS, Childhood Allergy and Asthma Study; COAST, Childhood Origins of Asthma Study; CCAAPS, Cincinnati Childhood Allergy and Air Pollution Study; CCCEH, Columbia Center for Children’s Environmental Health; EHAAS, Epidemiology of Home Allergens and Asthma Study; INSPIRE, Infant Susceptibility to Pulmonary Infections and Asthma Following RSV Exposure; IIS, Infant Immune Study; MAAP, Microbes, Allergy, Asthma, and Pets; TCRS, Tucson Children’s Respiratory Study; URECA, Urban Environment and Childhood Asthma; WHEALS, Wayne County Health, Environment, Allergy, and Asthma Longitudinal Study; WISC, Wisconsin Infant Study Cohort.

**Fig. 3.**
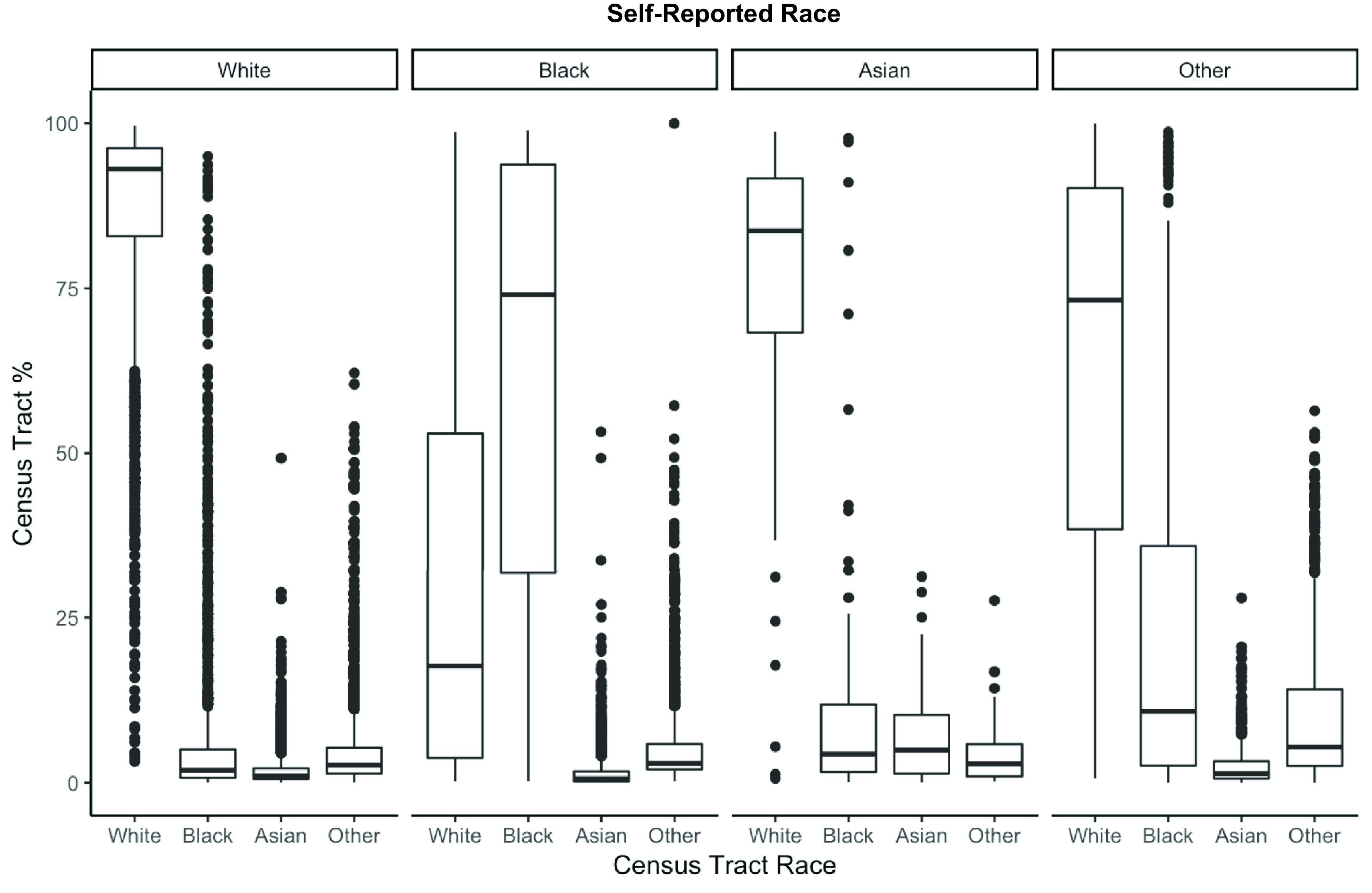
Comparison of census tract race to participants’ self-reported race.

The distribution of tract-level race data (%White, %Black, %Asian, %Other) according to participants self-reported race is presented in Figure 3. Overall, participants who reported being White or Asian race lived in census tracts with majority White populations, whereas participants who reported Black race resided in census tracts with greater variability in shares of White and Black populations (Figure 3). Additional comparisons of individual-level to neighborhood-level income and ethnicity are provided in the supplementary material.

## Discussion

Environmental exposures and the community in which they occur are significant causes of human disease, including asthma [7,14–17]. Disentangling the environmental and social exposures that contribute to health disparities necessitates geographically and culturally diverse studies. The methods described here make the characterization of community characteristics in a confidential yet standardized and reproducible manner more feasible for researchers and policy-makers. Importantly, our method is generalizable to additional types of geographic data, including polygon and point data, allowing other studies to customize and incorporate geocoding and geospatial analyses into their approach.

Our distributed geospatial approach offers some important advantages compared to existing methods, including reduced exposure misclassification, maintaining participant confidentiality, and reducing the need for geospatial expertise at each study site [1]. One existing and alternative method to maintain subject confidentiality is the alteration of participants’ geographic coordinates. Referred to as “geomasking” or “jittering,” this approach involves either a random shift in the location of subjects or a systematic transformation of the locations known only to the researchers [4]. However, this method may introduce errors or biases introduced due to the displacement of the participants’ actual location, particularly for analyses requiring an exact location. For example, the amount of geomasking required to make geographic datasets de-identified according to HIPAA standards may result in incorrect census tracts being linked to individual subjects resulting in exposure misclassification. An alternative approach to incorporating geospatial information into multisite studies is to obtain IRB approval and DUAs to share subjects’ identifiable information with a central site for geocoding and analysis. Challenges with this strategy include hesitation on the part of institutions to share identifiable information (e.g. addresses). Performing geospatial linkages at individual sites is another approach but may produce non-standardized and non-reproducible results due to the use of varying geocoding platforms, software, and dataset.

Our DeGAUSS method overcomes these limitations because the geospatial data and software are developed at a central site, ensuring that all individual study sites run the same software on identically constructed datasets. Importantly, our method also accounts for spatial, temporal, and informational changes in census tract boundaries and census data over the study period. Participants in CREW cohorts were born over nearly four decades, and, as detailed in the accompanying supplement, linking geocoded addresses with the appropriate census tract at the time of participant birth was critical to our approach. By including census tract boundaries and accompanying data from 1980, 1990, 2000, and 2010 in our DeGAUSS software, we were able to link study participants to the appropriate census tract and data for their date of birth. Of note, the DeGAUSS platform is flexible and amenable to additional geographic data and analyses, including derivation of nearby greenspace, distance to roadways and other locations, estimating gridded air pollution exposures, area-based indices of neighborhood deprivation, and others. Specific details and example uses are available on the DeGAUSS website [12,13].

As part of the NIH Environmental influences on Child Health Outcomes (ECHO) program, the objective of CREW is to understand the etiology of childhood asthma and determine environmental and genetic contributors. As such, CREW offers a unique opportunity to examine the significantly higher rates of asthma prevalence, hospitalization, and morbidity among children residing in households and neighborhoods with lower SES, as compared to White children residing in higher socioeconomic status (SES) households and communities [18–20]. Multiple influences contribute to these disparities including disproportionately higher exposure to air pollution, poor housing, limited access to care, and other adverse physical and toxicant contributors, and inherited factors may increase individuals’ sensitivity to these [21–25]. However, environmental exposures and inherited factors alone do not fully explain the observed health disparities. For that reason, hardships linked to poverty, including discrimination, stress, family turmoil, violence, instability, and others have been posited to play an important role in the social patterning of disease [26]. These social determinants of health may be separate from access to medical care and are important drivers not only in asthma morbidity, but also in a wide range of both adult and pediatric health outcomes, including mortality [27,28]. The importance of socioeconomic factors is highlighted by observations that disparities in health outcomes across strata of SES remain present within racial/ethnic groups [28].

Our methods and results should be considered, however, in the context of some limitations. Census tract boundaries alone may not accurately describe individuals’ neighborhood experience and the use of decennial census data rather than ACS data resulted in reduced temporal precision but allowed us to increase our historic reach to 1980. There are also limitations to the US Census data including a lack of specificity in certain variables such as ethnicity. For example, Hispanic participants in the TCRS and IIS in Tucson, AZ, likely differ in ancestry from Hispanic participants from cohorts in New York City, NY. Finally, our approach requires expertise at individual institutions to obtain patient or electronic health records.

In conclusion, we demonstrated the use of a distributed approach to conduct geospatial analyses for the 12 CREW cohorts that is also applicable to other multisite studies. Future applications of our method will include additional gridded data including land cover, air pollution models, and meteorological information. Future research to understand the etiology of childhood asthma will incorporate longitudinal residential locations throughout childhood and multilevel analyses of individual and neighborhood environments.

## Supplementary material

For supplementary material accompanying this paper visit http://dx.doi.org/10.1017/cts.2021.7. click here to view supplementary material
