# Supplementary material for: A distributed geospatial approach to describe community characteristics for multisite studies
Source: J Clin Transl Sci. 2021 Feb 5;5(1):e86. doi: 10.1017/cts.2021.7 (PMC8111696; doi:10.1017/cts.2021.7)
Supplement: Supplementary file 1 [file S2059866121000078sup001.docx]

**Supplementary Materials**

**Methods**

*The CREW Consortium*

CREW consists of 12 separate birth cohorts representing a diverse sample of children and their families residing throughout the U.S. in urban, suburban, and rural environments (Supplementary Table 1). Study eligibility varied by cohort and included children at normal risk for development of allergic disease and asthma and children at increased risk for allergic disease and asthma due to elevated environmental exposures and/or familial history of allergic disease and asthma (Supplementary Table 1). Birth years of cohort participants span nearly four decades, beginning in 1980 and continuing through the present (11). Study procedures were approved by the local institutional review board (IRB) at each participating cohort’s institution: University of Arizona, Boston University, Brigham and Women’s Hospital, University of Chicago, Cincinnati Children’s Hospital Medical Center, Columbia University, Henry Ford Health System, Johns Hopkins University, University of Wisconsin-Madison, Vanderbilt University Medical Center, Washington University, Marshfield Clinic Research Foundation, and University of Cincinnati. A data sharing protocol and a data use agreement (DUA) also was approved by the local IRB for each participating cohort. However, the DUA permitted only the sharing of limited data sets and did not allow for addresses or geocodes to be shared across institutions. Parents or legal guardians provided written, informed consent for their child’s study participation in each cohort, and all research was performed in accordance with relevant guidelines.

**Supplementary Table 1.** CREW Consortium Birth Cohorts and Study Populations

| **Cohort** | **Location(s)** | **Recruitment** | **Initial Enrollment** | **Study Population** |
| --- | --- | --- | --- | --- |
| Childhood Allergy / Asthma Study (CAS) | Detroit, MI | 1987-1989 | 835 | General |
| Childhood Origins of Asthma Study (COAST) | Madison, WI | 1998-2000 | 289 | High-risk^a^ |
| Cincinnati Childhood Allergy and Air Pollution Study (CCAAPS) | Cincinnati, OH | 2001-2003 | 762 | High-risk^a^ |
| Columbia Center for Children's Environmental Health (CCCEH) | New York City, NY | 1998-2006 | 727 | General |
| Epidemiology of Home Allergens and Asthma Study (EHAAS) | Boston, MA | 1994-1996 | 505 | High-risk^a^ |
| Infant Susceptibility to Pulmonary Infections and Asthma Following RSV Exposure (INSPIRE) | Nashville, TN | 2012-2014 | 1952 | General |
| Infant Immune Study (IIS) | Tucson, AZ | 1996-2004 | 482 | General |
| Microbes, Allergy, Asthma, and Pets (MAAP) | Detroit, MI | 2014-2016 | 120 | General |
| Tucson Children's Respiratory Study (TCRS) | Tucson, AZ | 1980-1984 | 1246 | General |
| Urban Environment and Childhood Asthma (URECA) | Baltimore, MD; Boston, MA; St. Louis, MO; New York City, NY | 2004-2006 | 609 | High-risk^a^ residing in urban areas with high rates of poverty |
| Wayne County Health Environment Allergy and Asthma Longitudinal Study (WHEALS) | Detroit, MI and Western Wayne County, MI | 2003-2007 | 1258 | General |
| Wisconsin Infant Study Cohort (WISC) | Marshfield, WI | 2013-2018 | 212 | General, rural residence, farm and non-farm |

^a.^ Parental history of asthma and/or allergic disease

*DeGAUSS*

DeGAUSS uses Docker, a software containerization platform, to wrap necessary software, system dependencies, and geospatial data in a standalone package that will work the same regardless of its host environment. Here, we created a custom DeGAUSS container to specifically meet the needs of the CREW consortium to link geocoded birth record addresses to appropriate (with respect to space and time) decennial U.S. Census tract and data. As CREW participants were born from the 1980s through 2018, this container included US. Census data and tract boundary files for 1980, 1990, 2000, and 2010. In addition, the DeGAUSS software contained R code (R Foundation for Statistical Computing: Vienna, Austria; 2014), including the geospatial packages sp (RS Bivand, Pebesma, & Gomez-Rubio, 2005), rgdal (Roger Bivand, Keitt, & Rowlingson, 2014) tigris (Walker, 2017), and tidycensus (Walker, 2018), to: 1) spatially join the geocoded birth record address to the census-tract polygon in which it was located and 2) append the census tract-level data for each geocoded birth address. At each study site, data managers geocoded their cohorts’ birth record addresses using either the available DeGAUSS geocoder or other geocoding software (if addresses were previously geocoded). The CREW DeGAUSS container required only a .csv file of geocoded locations and end-user specification of which census year (1980, 1990, 2000, 2010) to use for calculations. Additional information, including code and example uses are available at the DeGAUSS website and specifically for the CREW container (12, 13)

*Geocoding and Census Year Linkage*

Prior to implementing the DeGAUSS image, each cohort abstracted, organized, and geocoded the birth addresses for participants at their site. Cohorts (n = 3) with previously geocoded and validated birth record addresses using ArcGIS (ESRI, Redlands, CA) or other geocoding software used these coordinates while the remaining cohorts geocoded their participants birth record addresses using an available DeGAUSS geocoder image (5, 12). The DeGAUSS geocoder is based on street range interpolation and therefore addresses not able to be geocoded at the street or address level were removed prior to analyses. CREW cohort participants born from 1976 – 1985 were linked to 1980 census tracts and data, while participants born between 1986 – 1995, 1996 – 2005, and 2006 – 2017 were linked to 1990, 2000, and 2010 census tracts and data, respectively.

*U.S. Census / American Community Survey data*

Census tract-level information included in the DEGAUSS container and linked to all study participants birth record address comprised the following variables: census tract area (km^2^), total population, population density (population per km^2^), median household income (US Dollars (USD)), percentage (%) of population on assisted income, % below the poverty level, % White population, % Black population, % Asian population, % other (non-White, non-Black, non-Asian) population, % Hispanic population, highest education attainment (% without high school diploma, % with high school diploma, % bachelor’s degree), % occupied housing, % owner occupied housing, median gross rent (USD), median housing value (USD), housing density (housing units per km^2^), % female head of household, % single parent head of household, and % unemployed. All variables reported in USD (median household income, median gross rent, and median housing value) were inflation-adjusted to 2012 USD using the Bureau of Labor Statistics' Annual Average Consumer Price Index Research Series (1947 to 2017) (<https://www.census.gov/topics/income-poverty/income/guidance/current-vs-constant-dollars.html>).

Linkage with the correct census tract boundary was a crucial consideration to our approach. Corresponding to the increase in the U.S. population from 226.5 million in 1980 to 308.7 million in 2010, the number of census tracts increased from 46,728 in the 1980 census to 74,001 tracts in the 2010 census. Therefore, census tract polygon boundaries in ESRI shapefile format (for 1980, 1990, 2000, and 2010) were downloaded from the National Historic GIS (NHGIS) data service. The 1980, 1990, and 2000 files differ from the boundaries provided by the Census Bureau in that they were manually corrected by NHGIS based on 2008 TIGER/Line files to correct for geographic inaccuracies (called “conflated census tract files” by NHGIS). These files were were downloaded, merged with the tabular variables by census tract FIPS codes, and loaded into our DeGAUSS software, thereby allowing for proper temporal census tract variable assignment for each member of each cohort. In addition, the information collected from the 1980 through 2010 decennial census has changed, including the number and type of questions asked, variable names, and variable reporting methods. In order to best compare our indicator variables over time we had to take several steps. First, to supplement the 2010 decennial census data, variables were downloaded from the 5-year American Community Survey (ACS) for the years 2008 – 2012. Second, to ensure consistency and enable exploration of our indicator variables we created a matrix with columns for the years 1980, 1990, 2000, and 2010 and rows for each of the variables: 63 for 1980, 92 for 1990, 164 for 2000, and 150 for 2010 (132 of these 2010 variables from the ACS). Exact variable names and descriptions from the U.S. Census were entered into this matrix and notes of our own as we examined the variables and made comparisons. Third, to enable consistent comparison of the variables reported in USD (median household income, median gross rent, and median housing unit value) we created additional inflation adjusted variables. As the 2010 ACS data is in 2012 dollars, we used the Bureau of Labor Statistics' (BLS) Annual Average Consumer Price Index Research Series (1947 to 2017) to convert dollar amounts from the 1980, 1990, and 2000 datasets to their corresponding amounts in 2012.

*Comparison of Census Tract-level to Self-reported Race, Ethnicity, and Income*

The distribution of Census tract-level race data (% White population, % Black population, % Asian population, and % other) was plotted according to self-reported race categories (White, Black, Asian, or other). Similarly, the percentage of Hispanic population in the census tract was compared to self-reported ethnicity. Household income was collected by individual cohorts using different income categories. Self-reported household income at time of birth was categorized as ‘high’ and ‘low’ based on the available data for each cohort (< $40,000 / > $40,000 for CAS, CCAAPS, CCCEH, COAST, INSPIRE, MAAP, URECA, WHEALS; < /> $30,000 for EHAAS; < / > $35,000 for IIS and TCRS; and < / > $25,000 for WISC). The distribution of census tract median household income was described for each self-reported category (high / low) using box-and-whisker plots for the overall CREW consortium.

**Results**

A summary of census tract-level housing, disadvantage, and socioeconomic data is provided in Supplementary Table 2. Cohorts whose participants resided in tracts with the lowest proportion of occupied housing, on average, included URECA-Baltimore (77%) and URECA-St. Louis (78%). On average, CREW participants resided in tracts at birth where 17% of households were headed by females, 21% of households had a single parent, and 10% of the population was unemployed (Supplementary Table 2). Participants in the URECA cohort resided in tracts with the highest rates of the population with single-parent households while the cohorts in Wisconsin (COAST and WISC) generally had the lowest percentage of these indicators.

**Supplementary Table 2.** Summary of Census Tract-level Income, Housing, and Measures of Disadvantage for CREW Participants at Birth Record Address

|  | **Census Variable (mean, SD)** | | | | | |
| --- | --- | --- | --- | --- | --- | --- |
|  | **Housing** | | | **Disadvantage** | | |
| **Cohort** | **% Occupied Housing** | **Median Housing Value (2012 USD)** | **Median Gross Rent (2012 USD)** | **% Female Head of Household** | **% Single Parent Household** | **% Unemployed** |
| CREW | 89 (9) | 184,300 (135,961) | 838 (285) | 17 (12) | 21 (14) | 10 (8) |
| CAS | 85 (16) | 156,183 (72,267) | 914 (305) | 10 (5) | 13 (6) | 6 (3) |
| CCAAPS | 93 (5) | 162,278 (62,725) | 768 (289) | 14 (11) | 18 (12) | 5 (6) |
| CCCEH | 92 (5) | 293,933 (245,154) | 743 (135) | 34 (7) | 41 (7) | 18 (6) |
| COAST | 94 (5) | 206,643 (59,954) | 874 (126) | 8 (3) | 12 (4) | 4 (2) |
| EHAAS | 84 (10) | 420,495 (175,030) | 1,249 (320) | 12 (8) | 16 (10) | 5 (3) |
| IIS | 91 (6) | 164,425 (80,148) | 916 (365) | 11 (5) | 15 (6) | 5 (3) |
| INSPIRE | 90 (5) | 159,836 (77,293) | 808 (246) | 15 (9) | 20 (10) | 10 (6) |
| MAAP | 92 (5) | 175,675 (85,716) | 1,036 (354) | 12 (5) | 16 (6) | 11 (5) |
| TCRS | 92 (4) | 161,028 (57,600) | 767 (214) | 9 (4) | 6 (3) | 6 (2) |
| URECA - Baltimore | 77 (13) | 105,776 (61,364) | 764 (258) | 35 (11) | 42 (12) | 18 (8) |
| URECA - Boston | 90 (5) | 321,040 (100,315) | 998 (314) | 30 (12) | 37 (13) | 14 (7) |
| URECA - New York | 91 (9) | 422,084 (293,362) | 758 (308) | 34 (10) | 41 (11) | 15 (6) |
| URECA - St. Louis | 78 (11) | 87,492 (42,447) | 673 (147) | 31 (10) | 37 (11) | 18 (8) |
| WHEALS | 87 (11) | 116,139 (78,886) | 830 (253) | 24 (12) | 30 (14) | 15 (11) |
| WISC | 89 (12) | 140,318 (26,496) | 618 (79) | 7 (2) | 12 (2) | 6 (2) |

CREW- Children's Respiratory and Environmental Workgroup, CAS - Childhood Allergy and Asthma Study, COAST - Childhood Origins of Asthma Study, CCAAPS - Cincinnati Childhood Allergy and Air Pollution Study, CCCEH - Columbia Center for Children's Environmental Health, EHAAS - Epidemiology of Home Allergens and Asthma Study, INSPIRE - Infant Susceptibility to Pulmonary Infections and Asthma Following RSV Exposure, IIS - Infant Immune Study, MMAP - Microbes, Allergy, Asthma, and Pets, TCRS - Tucson Children's Respiratory Study, URECA - Urban Environment and Childhood Asthma, WHEALS - Wayne County Health, Environment, Allergy, and Asthma Longitudinal Study, WISC - Wisconsin Infant Study Cohort

*Comparison of individual-level to tract-level data*

All but one cohort (CCCEH) provided self-reported, individual-level household income, ethnicity, and race data to compare to analogous tract-level data. The distribution of tract-level median household income by self-reported household income category (high / low as defined above) is presented in Supplementary Figure 1. The majority of participants reporting household income <$40,000 resided in census tracts having median household incomes <$50,000. Among participants reporting having household incomes >$40,000, the variability of tract-level median household income was greater, and the average tract-level median household income was significantly higher. CREW participants who reported being non-Hispanic generally lived in census tracts with low Hispanic populations, while participants who reported having Hispanic ethnicity lived in census tracts with greater Hispanic populations.

**Supplementary Figure 1.** Distribution* of Tract-level Median Household Income by Self-reported Household Income (A) and Census Tract-level Ethnicity by Self-reported Ethnicity (B)

* Box represents the interquartile range (IQR); median designated by vertical line; whiskers extend +1.5 times the IQR; dots represent outliers > / < 1.5* IQR


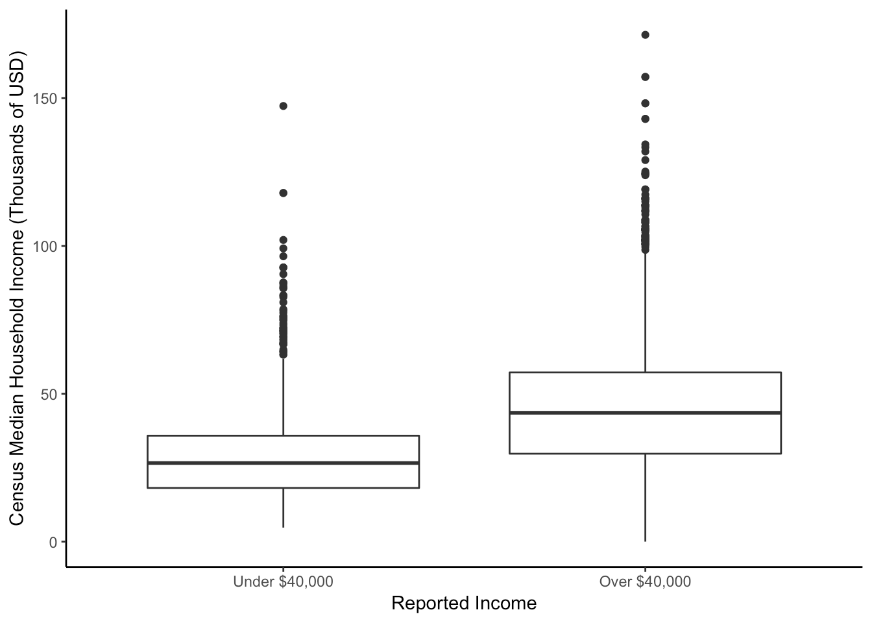

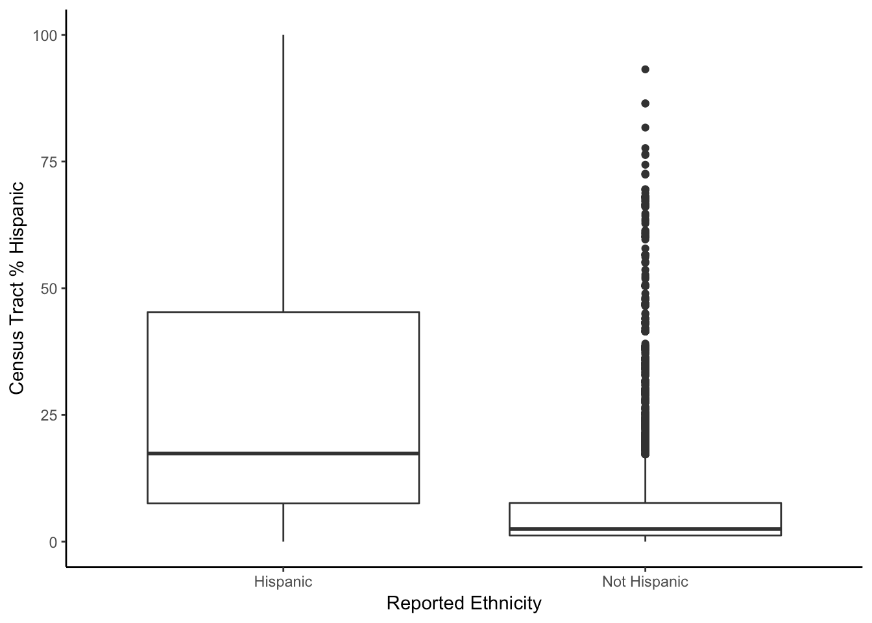


**A.**

**B.**
